# Entrepreneurial mental health in the wake of COVID-19 in China with an emphasis on attention deficit hyperactivity disorder (ADHD) and dyslexia analysis

**DOI:** 10.1038/s41598-024-56981-8

**Published:** 2024-03-19

**Authors:** Yijun Fan, Yuanzhe Li, Zhengyuan Dong, May Ong, James Hope

**Affiliations:** 1 Department of Chinese Language Studies (CHL), The Education University of Hong Kong New Territories, Hong Kong, SAR China; 2 School of Civil and Environmental Engineering, University of Auckland, Auckland, 1010 New Zealand; 3 College of Design and Engineering, National University of Singapore, Singapore, 117575 Singapore; 4 School of Civil, Aerospace and Mechanical Engineering, University of Bristol, Bristol, BS8 1QU UK; 5 Stirling Management School, Singapore Institute of Management, Singapore, 599491 Singapore; 6 Sustainability Design Institution, China Academy of Art, Hangzhou, 310002 China

**Keywords:** Entrepreneurship in China, Mental health impact, COVID-19 pandemic, Attention deficit hyperactivity disorder (ADHD), Dyslexia, Health care, Health occupations

## Abstract

The COVID-19 pandemic has precipitated a global mental health crisis, with a particularly pronounced impact on the entrepreneurial sector. This paper presents a comparative analysis of mental health challenges among entrepreneurs in China during the pandemic, with a specific focus on attention deficit hyperactivity disorder (ADHD) and Dyslexia. The study assesses the prevalence of ADHD and dyslexia symptoms among established and emerging entrepreneurs in China, finding notable occurrences within this group. The research also examines the self-care practices of these entrepreneurs, shedding light on their approaches during the pandemic period. The findings highlight a complex interplay between mental health issues and entrepreneurial activities, suggesting that certain ADHD and dyslexia traits may offer unexpected benefits in the entrepreneurial realm. These insights are critical for developing supportive frameworks that leverage the strengths of neurodiverse entrepreneurs while mitigating associated challenges, especially in a post-pandemic economic landscape. The study concludes with policy and practice recommendations to bolster the wellbeing and resilience of entrepreneurs facing the multifaceted impacts of the pandemic.

## Introduction

The COVID-19 pandemic, caused by the SARS-CoV-2 virus, was first identified in Wuhan, China, in December 2019. It quickly spread throughout China, and by January 2020, it had become a global health crisis. The Chinese government implemented strict measures to contain the spread of the virus, including lockdowns, travel restrictions, and mass testing. These measures were effective in reducing the spread of the virus in China, but the pandemic continued to affect the country's economy and mental health^1^. The pandemic had a significant impact on mental health in China, with people experiencing high levels of anxiety, depression, and stress. The strict measures implemented to contain the spread of the virus, including lockdowns and social distancing, had a profound impact on people's daily lives, work, and relationships. Many people lost their jobs, and the economic uncertainty and financial stress contributed to the mental health burden^2^. The pandemic also disrupted mental health services, making it difficult for people to access care and support. Entrepreneurs were also affected by the pandemic, with many facing significant challenges in their businesses^3^. The uncertainty and economic disruption caused by the pandemic made it difficult for entrepreneurs to plan and make decisions, leading to high levels of stress and anxiety. The pandemic also highlighted the importance of mental health and self-care for entrepreneurs, as they struggled to balance the demands of their businesses with their own wellbeing.

The COVID-19 pandemic has profoundly impacted China, causing widespread economic disruption and negative effects on mental health. Strict containment measures implemented by the Chinese government, including lockdowns and social distancing protocols, significantly altered daily life and took a toll on wellbeing. These restrictions hindered entrepreneurs' abilities to operate, plan, and make informed decisions, leading to heightened anxiety and uncertainty. However, the crisis also catalyzed China to prioritize mental health to an unprecedented degree^4^. In March 2020, the government issued guidelines for mental health services during the pandemic, emphasizing the need for psychological support. The state also initiated campaigns promoting self-care and help-seeking behaviors to bolster resilience^5^.

This study investigates how established entrepreneurs (those running businesses for over 3 years) and nascent entrepreneurs (in the process of starting ventures) navigated these challenges. The purpose of comparing established and nascent entrepreneurs allows the research to capture a broader spectrum of the entrepreneurial experience, particularly how different stages of entrepreneurship correlate with mental health, well-being, and the ability to cope with challenges like those presented by the COVID-19 pandemic. Established entrepreneurs have been in operation for longer, which usually indicates that they have seen different phases of expansion and stability. Conversely, nascent entrepreneurs are those that are just beginning their firm and are dealing with a variety of issues like funding acquisition, managing early-stage growth, and business validation. The COVID-19 pandemic may have had different effects on established and nascent entrepreneurs. While established entrepreneurs may have hurdles in maintaining and adjusting an existing business model, new entrepreneurs may be concerned about the timing of market entry and the feasibility of their business ideas during a crisis. The contrast in business maturity stages can yield insights into how the experience and pressures of entrepreneurship affect mental health differently. It focuses specifically on entrepreneurs exhibiting symptoms of attention-deficit/hyperactivity disorder (ADHD) or dyslexia. Previous research shows ADHD and dyslexia symptoms are disproportionately prevalent among entrepreneurs compared to the general populace^6^. The analysis concentrates on businesses founded after 2018 to ensure relevance to the current climate and capture recent adaptive responses. The pandemic likely exacerbated existing mental health issues facing entrepreneurs with ADHD or dyslexia^7^. Studies demonstrate 43% of Chinese citizens reported worsened anxiety and depression due to COVID-19 hardships. Entrepreneurs likely faced similar struggles, with adversity and uncertainty potentially worsening pre-existing conditions^2–4,8^. The findings indicate a prevalence of ADHD and dyslexia symptoms among entrepreneurs, with nascent entrepreneurs reporting lower levels of wellbeing than their established peers. Entrepreneurs with ADHD or dyslexia symptoms also showed a trend of reduced life satisfaction relative to those without such symptoms. This approach allows for an understanding of mental health within the entrepreneurial sector in China, contributing to a body of knowledge that could inform future research and targeted interventions. Moreover, this comprehensive analysis also provides critical insights into the complex interplay between entrepreneurship, mental health, and the unique challenges posed by the pandemic. Entrepreneurs are often at the forefront of innovation and economic development, and safeguarding their mental health is critical to sustaining their valuable contributions to society. As such, this research can catalyze the development of targeted support mechanisms and underscores the need for targeted support and resources for entrepreneurs, particularly those grappling with mental health issues, to foster a more resilient and mentally healthy entrepreneurial ecosystem.

## Literature review—challenges, coping strategies, and theoretical frameworks

### Mental health and entrepreneurship in post-pandemic period of China

The COVID-19 pandemic delivered an economic shock to China, with widespread business disruptions and financial hardship for entrepreneurs. In response, the government implemented relief policies, including financial subsidies and loans, to bolster entrepreneurial resilience^9^. The crisis also accelerated digitization, opening opportunities amid adversity^3,10^. However, a national study found heightened anxiety and depression among entrepreneurs, linked to uncertainty, isolation, and financial strains of the pandemic. Entrepreneurs with attention-deficit/hyperactivity disorder (ADHD) or dyslexia likely faced compounded struggles. Research shows some ADHD traits align with entrepreneurial behaviors, like adaptability and impulsivity enabling seizeing of opportunities. However, the stress of navigating a rapidly digitizing climate may exacerbate ADHD distractedness and impulsivity. Similarly, dyslexia poses barriers to transitioning online. This intersection of business and mental health challenges calls for specialized support catering to this group's needs^11^.

A growing body of research reveals connections between ADHD symptomatology and entrepreneurial tendencies. As outlined in Table 1, certain hallmark characteristics of ADHD appear to confer strengths in the realm of business leadership and strategic decision-making^12^. For instance, behavioral tendencies like hyperfocus, proclivity for multitasking, comfort with risk, and heightened impulsivity carry advantages in recognizing opportunities, mobilizing resources, and navigating uncertainties. However, ADHD symptoms also introduce potential impediments, especially when exacerbated by external stressors. Problems with organization, communication, and emotional regulation can hamper productivity and relationships essential for entrepreneurial success^13^. As such, it is vital to consider the double-edged nature of the ADHD-entrepreneurship interface when examining mental health challenges faced by business founders, particularly amid destabilizing global events like the COVID-19 pandemic^10,14^. Targeted interventions that mitigate ADHD difficulties while retaining helpful orientations could powerfully equip these entrepreneurs for the complexities of leadership^15^. For example, coaching in structure and priority-setting can augment productivity, while training in mindful stress management can enhance focus. Such tailored supports not only aid individual resilience, but also strengthen the wider entrepreneurial ecosystem by setting up a critical demographic for sustenance and growth^16^.

**Table 1:** Similarities between ADHD symptoms and entrepreneurial behaviours.

ADHD traits	Corresponding entrepreneurial behaviours
Easily distracted	Entrepreneur's adaptability in shifting focus to new business opportunities
Daydreamer, visual thinker	Capacity for goal visualization and strategic planning
Disorganized	Agile in responding to changing business landscapes
Socially undeveloped	Openness to learning from failures and feedback
Initiates multiple projects	Multitasking and versatility in project management
Impulsive	Rapid decision-making and seizing of business opportunities
Prone to act without considering consequences	Risk-taking propensity for business growth
Low self-esteem	Humility, which can foster team collaboration
Hyperactive	Sustained energy for business development tasks
Inattentive	Tendency to be involved in long-term strategic thinking
Poor verbal communication	Preference for direct, action-oriented communication
Distorted time perception	Deep focus and dedication to work-related tasks
Intrusive	Proactive customer engagement and service
Hands-on learner	Practical approach to business management and problem-solving

Amid the pandemic, entrepreneurs with ADHD or dyslexia may face intensified challenges. The added stressors of navigating a rapidly digitizing market can exacerbate ADHD-related distractibility and impulsivity, while dyslexia can make the transition to digital platforms particularly demanding. This intersectionality of entrepreneurial stress and neurodevelopmental disorders calls for specialized mental health support tailored to the unique needs of this demographic^17^. The study by Haltiwanger et al. also identifies coping strategies beneficial in mitigating the pandemic's impact on entrepreneurs' mental health. These include developing robust social networks, engaging in physical activities, and maintaining a positive outlook^9,14^.

Studies identify beneficial coping strategies for entrepreneurs, including robust social connections, physical activity, and maintaining positivity. As China progresses post-pandemic, continuous emphasis on entrepreneurial mental health will be vital, particularly for those managing neurodevelopmental disorders alongside business leadership^18^. Prioritizing psychological wellness and adaptive strategies will nurture resilience in this demographic and strengthen the entrepreneurial ecosystem^19^.

### Theoretical frameworks and concepts for ADHD and dyslexia study

The advent of the COVID-19 pandemic has precipitated economic and psychological upheaval, disproportionately affecting entrepreneurs. The resultant lockdowns and economic instability have amplified stress, with entrepreneurs in China experiencing acute mental health challenges. The prevalence of anxiety and depression among Chinese adults, particularly entrepreneurs, has surged, a phenomenon substantiated by the Chinese Association of Mental Health^20^. The 'stress process model' posits that the stressors intrinsic to entrepreneurship, coupled with pandemic-induced financial and familial strains, can precipitate mental health crises^10^.

Within this milieu, the nuances of ADHD symptomatology in entrepreneurs have garnered attention. The distinction between clinically diagnosed ADHD and a broader spectrum of ADHD symptoms is critical. While clinical ADHD encompasses a set of symptoms severe enough to impair functioning significantly, subclinical ADHD includes traits and behaviors characteristic of ADHD that do not meet the full diagnostic criteria as stipulated by DSM-5 or ICD-10^13,14^. Entrepreneurship studies frequently focus on this broader, subclinical symptomatology, acknowledging that many adults with ADHD traits successfully navigate various settings, possibly due to developed compensatory strategies. This spectrum approach allows researchers to explore how ADHD traits, ranging from mild to moderate, might shape entrepreneurial behaviors and outcomes without the constraints of a clinical diagnosis^6–8^.

ADHD, characterized by hyperactivity, impulsivity, and inattention, may present challenges in entrepreneurship, such as issues with time management, organization, and sustained focus, potentially hindering business performance. Similarly, dyslexia, a learning disorder that impedes reading and writing capabilities, can make it arduous for entrepreneurs to engage with written materials essential for business operations, like contracts and business plans^13^. Despite these impediments, current research also highlights potential strengths inherent in individuals with ADHD and dyslexia, which may confer entrepreneurial advantages. For instance, the creativity often associated with ADHD and the visual-spatial aptitude linked to dyslexia could provide a competitive edge in innovation-driven sectors^6,7^.

## Methodology

The study deployed a cross-sectional survey design to assess the psychological and entrepreneurial characteristics of established and nascent entrepreneurs in the post-pandemic landscape of China. The survey distribution targeted individuals associated with the Business School of Shanghai Jiaotong University, including MBA students, alumni, and members of the Undergraduate Innovation and Entrepreneurship Club, as well as through expansive online social media outreach^4^. Ensuring voluntary participation, respondents were given the option to submit contact details for future dissemination of the study's findings and feedback^4–7^.

Out of 647 responses gathered within two months in Q3 2022, a refined selection of 350 responses from established entrepreneurs and 288 from nascent entrepreneurs were deemed analytically viable. The study honed in on businesses formed after 2018, resulting in a focused sample size comprising 173 established and 244 nascent entrepreneurs. The survey solicited data spanning demographics, educational levels, industry experience, previous entrepreneurial ventures, and familial business background. Within this cohort, the mean age was 33.3 years, split between established entrepreneurs at 37.1 years and nascent entrepreneurs at 30.6 years. The gender distribution featured 66% male and 29% female, with 5% choosing not to specify. Measurement instruments integrated into the survey included the Adult ADHD Self-Report Scale (ASRS-6) and the Adult Dyslexia Checklist, tailored to detect indications of ADHD and dyslexia symptoms^13,14^. Additionally, a life satisfaction scale was employed to gauge overall wellbeing.

Aligned with the Span et al.^21^ three-factor model of ADHD and the DSM-IV two-factor classification of ADHD symptoms, the study's statistical analysis bifurcated ADHD into dimensions of inattention and hyperactivity–impulsivity. Descriptive statistics distilled participant characteristics and symptom prevalence, while chi-square tests and t-tests compared mental health symptoms and entrepreneurial behaviors. Logistic regression models probed the association between ADHD dimensions—specifically inattention and hyperactivity–impulsivity—and entrepreneurial behaviors^22^. These models controlled for potential confounders such as age, gender, and years of experience, providing a structured approach to analyze the likelihood of entrepreneurial behavior in connection with ADHD dimensions^23^. As indicated by Fisher and Barkley^24^, a Multivariate Analysis of Variance (MANOVA) was deployed to detect significant disparities in self-care behaviors and life satisfaction across both entrepreneur categories and among those exhibiting varying degrees of ADHD or dyslexia symptoms. This choice of analysis was advantageous for its capacity to handle multiple dependent variables and to reveal complex interrelations within the data^24^. The application of MANOVA enables a more detailed understanding of the behavioral patterns that distinguish entrepreneurial groupings, providing information into possible stress points and areas for proactive support. Effect size, crucial for interpreting the practical significance of findings, was calculated using Cohen's d, particularly when analyzing mean differences between groups. Through such detailed research, we can better assist the entrepreneurial ecosystem, creating a more conducive climate for business innovation and growth in a post-pandemic period.

### Ethical approval

All procedures performed in studies involving human participants were in accordance with the ethical standards of both National University of Singapore (NUS) and University of Auckland (UoA) Research Committee. The study protocol was reviewed and approved by the NUS Research Committee prior to the commencement of the study. Written informed consent was obtained from all participants before they were included in the study. Confidentiality and anonymity were maintained throughout the study, and the data collected were used only for research purposes.

### Informed consent

Informed consent was obtained from all individual participants included in the study.

## Results and discussion

### Prevalence of ADHD and dyslexia symptoms among entrepreneurs

The behavioral patterns of entrepreneurs often mirror the symptoms observed in ADHD patients. The pervasive impact of the COVID-19 pandemic has raised concerns about the mental well-being of individuals with ADHD and dyslexia^3^. Researchers believe that the pandemic's aftermath could synergistically exacerbate the risk of developing ADHD and dyslexia among entrepreneurs^25^. This is particularly concerning given that entrepreneurs are inherently subjected to high levels of stress and uncertainty. Regarding lifestyle engagements, a significant portion of participants indicated high levels of engagement in faith-related activities. Conversely, trends toward lower engagement were observed for alcohol consumption and smoking, with several participants reporting moderate to critical levels of engagement. These variations suggest that individual engagement levels vary across activities, some with more consistency than others^26^. This data could be instrumental in pinpointing areas where individuals might benefit from increased engagement, thereby informing public health initiatives to foster healthier lifestyles. In alignment with current assessment criteria for ADHD and dyslexia, this study invited entrepreneurs to self-assess their behavior habits, information processing capabilities, motivation, focus, and self-awareness. Subsequent data analysis revealed a noticeable prevalence of ADHD and dyslexia symptoms among the entrepreneurial cohort.

**Figure 1:**
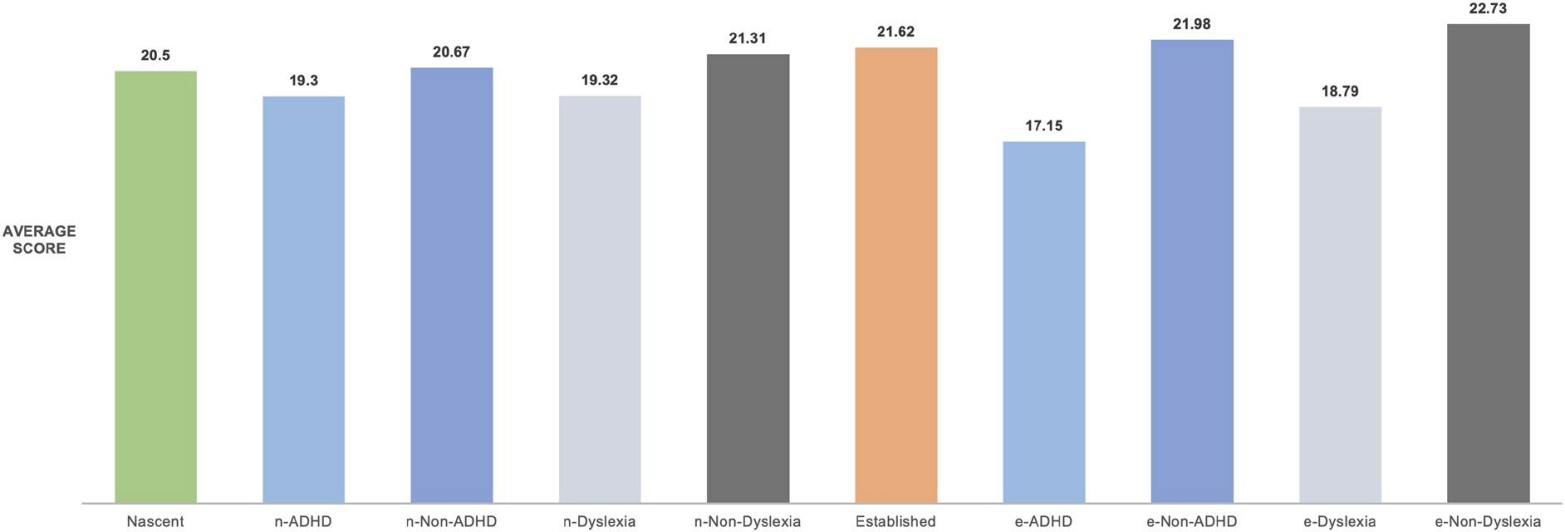
Comparison of wellbeing score of different groups of all entrepreneurs (categorized by nascent and established).

**Figure 2:**
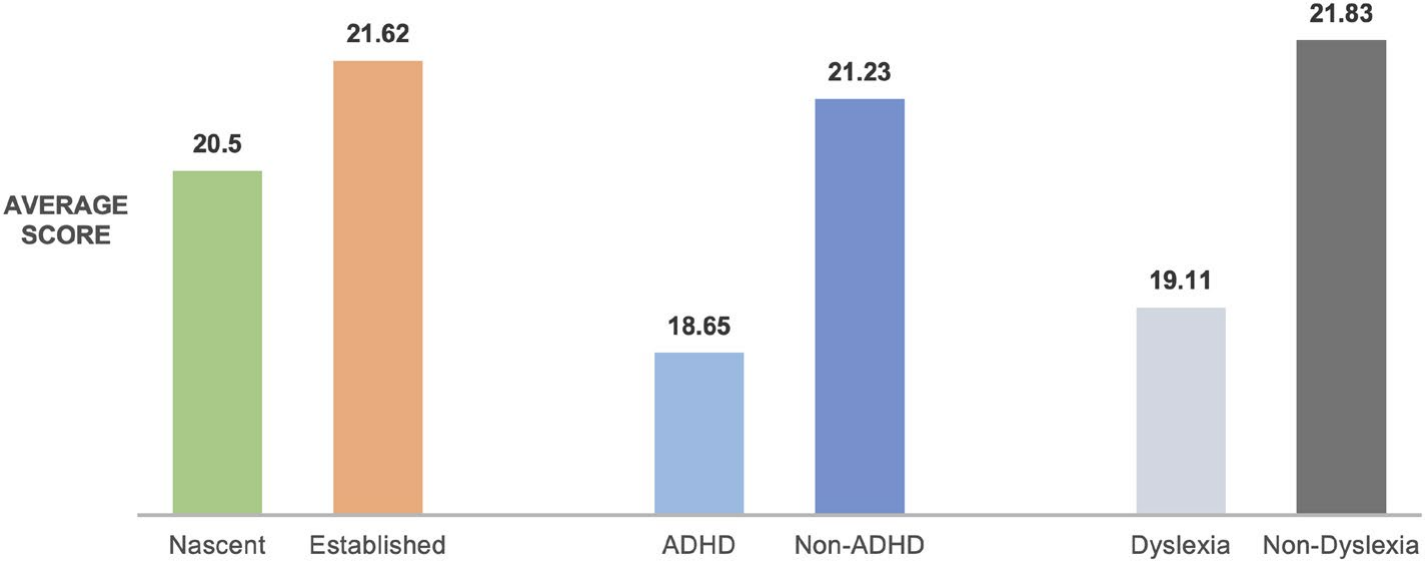
Comparison of wellbeing score of different groups of all entrepreneurs (categorized by ADHD and Dyslexia).

Among 173 established entrepreneurs, 13 were identified as having a high likelihood of ADHD, representing 8% of the surveyed group. For nascent entrepreneurs, 29 out of 244 exhibited ADHD symptoms, approximately 12%. This suggests a marginally higher incidence of ADHD among nascent entrepreneurs, potentially heightened by the stresses of the pandemic (Table 2). The study also compared the well-being scores among entrepreneurs with and without ADHD or dyslexia symptoms. Established entrepreneurs with ADHD symptoms had an average well-being score of 17.15, markedly lower than the 21.98 for those without symptoms. Similarly, nascent entrepreneurs with ADHD symptoms scored an average of 19.30 compared to 21.31 for those without (Fig. 1). The disparity was more pronounced among dyslexic entrepreneurs, with nascent individuals scoring 19.30 and established scoring 18.79, versus 22.73 for their non-dyslexic counterparts (Fig. 2). These findings suggest that COVID-19-related stress and uncertainty may exacerbate the negative impact of ADHD and dyslexia symptoms on entrepreneurs' mental wellbeing^27^.

**Table 2:** Established and nascent ADHD & dyslexia number of the participants.

	ADHD		Non-ADHD	Dyslexia	Non-Dyslexia
	AD	HD			
Established n = 173	4	9	160	48	125
Nascent n = 244	9	20	215	85	159

The differentiation of Attention Deficit (AD) and Hyperactivity Disorder (HD) symptoms is further studied in the following chapters based on the diagnostic criteria from the DSM-IV, which categorizes ADHD into two principal components: inattention, which encapsulates AD symptoms, and hyperactivity–impulsivity, which encompasses HD symptoms^13^. In the realm of Attention Deficit symptoms, individuals may find it challenging to complete the less stimulating aspects of a task or project, particularly when it comes to wrapping up final details. They often experience a diminished capacity to organize effectively, which is critical when tasks demand a structured approach. Moreover, these individuals might struggle with memory, frequently forgetting appointments or commitments, indicative of a reduction in their ability to retain and recall information. On the other hand, those with Hyperactivity Disorder symptoms exhibit an inability to remain still, often fidgeting or squirming when required to sit for long periods, suggesting a decrease in their impulse control^28^. They may also feel an incessant drive to be active, as if propelled by an internal motor, which points to an increased level of psychological stress and an inability to regulate their activity levels^29^.

### Attention deficit (AD) and hyperactivity disorder (HD) symptom and wellbeing regression analysis

The output of a linear regression model for Nascent and Established entrepreneurs is provided, where the presence of “Attention Deficit (AD) symptoms = 1” versus “Hyperactivity Disorder (HD) symptoms = 0” is modeled as the dependent variable. Independent variables in the model include lifestyle factors such as sleep duration, exercise frequency, yoga practice, healthy diet, religious engagement, alcohol consumption, and smoking.

The core objective of this regression analysis is to elucidate the interrelationships between these daily lifestyle factors and AD/HD symptom prevalence among entrepreneurs. This information is especially important in the context of entrepreneurship during the pandemic, which is characterized by increased stress and uncertainty. Understanding how modifiable activities might worsen or ameliorate AD/HD symptoms is critical for developing targeted wellness strategies. The observed challenges and stressors associated with entrepreneurship during the pandemic highlight the need for targeted support and interventions to enhance mental well-being among entrepreneurs. For instance, encouraging improved sleep hygiene, exercise, and a balanced diet could be useful tactics for entrepreneurs experiencing symptoms of AD/HD symptoms, while also considering possible therapies to address substance use. As such, delineating how certain modifiable behaviors may mitigate or contribute to attention deficit and hyperactivity issues can inform targeted wellness initiatives^18,19^.

For established entrepreneurs (Table 3), increased sleep duration exhibits a positive and marginally significant (p = 0.076) coefficient. This points to a potential link between greater sleep and attentional difficulties, perhaps reflecting an underlying neurological imbalance. However, other variables like exercise, diet, and yoga demonstrate no significant relationships, positive or negative^30^. Intriguingly, increased religious engagement appears moderately protective, evidenced by its negative coefficient, although statistical significance is lacking.

**Table 3:** The output of a linear regression model of AD & HD for established group.

Wellbeing variable	Coefficient	Std. error	t-value	P >|t|	95% conf. Interval (lower)	95% conf. interval (upper)
Sleep	0.332	0.149	2.23	0.076	− 0.051	0.715
Exercise	− 0.118	0.346	− 0.34	0.747	− 1.006	0.77
Yoga	1.283	2.044	0.63	0.558	− 3.97	6.536
Healthyfood	− 0.075	0.137	− 0.54	0.609	− 0.428	0.278
Religion	− 1.212	1.508	− 0.8	0.458	− 5.088	2.663
Alcohol	0.32	0.555	0.58	0.589	− 1.107	1.747
Smoke	− 0.205	0.284	− 0.72	0.503	− 0.936	0.526
_cons	− 0.256	0.556	− 0.46	0.665	− 1.686	1.174

Among nascent entrepreneurs (Table 4), the connection between sleep and Attention Deficit symptoms is further pronounced, with a large, positive, and statistically significant (p = 0.048) regression coefficient. This suggests that for emerging business owners, ensuring adequate sleep quantity and quality should be a priority to maintain focus and organization. Additionally, while not meeting significance thresholds, alcohol consumption demonstrates a positive correlation, whereas baseline AD/HD symptomology (the constant term) trends negative.

**Table 4:** The output of a linear regression model of AD & HD for nascent group.

Wellbeing variable	Coefficient	Std. error	t-value	P >|t|	95% conf. interval (lower)	95% conf. interval (upper)
Sleep	3.981	2.010	1.981	0.048	0.042	7.920
Exercise	0.330	0.844	0.390	0.696	− 1.326	1.985
Yoga	0.895	1.117	0.801	0.423	− 1.295	3.084
Healthyfood	− 0.257	1.239	− 0.208	0.835	− 2.685	2.170
Religion	− 0.010	0.861	− 0.012	0.991	− 1.697	1.676
Alcohol	1.677	2.096	0.800	0.424	− 2.430	5.785
Smoke	− 0.448	1.475	− 0.304	0.761	− 3.339	2.443
const	− 14.661	9.135	− 1.605	0.108	− 32.565	3.243

The raw data tables of ADHD symptoms among the entrepreneur subgroups allows for some additional quantitative analysis and insights (Table S1) regarding the prevalence and intensity of Attention Deficit and Hyperactivity markers across established and nascent entrepreneurs. Established founders exhibit particular difficulty with remembering obligations, avoiding thoughtful work, excessive activity, and fidgeting. For nascent owners, remembering appointments, organization challenges, restlessness, and distraction are most common. "Often/very often" responses show broadly comparable severity between groups. Notably, both subgroups report higher symptom totals for Hyperactivity versus Inattention, aligned with the view of heightened drive and energy as entrepreneurial traits. Key AD challenges like disorganization and memory issues may impact vital business skills like prioritization, time management, and strategic thinking. Meanwhile, restlessness and impulsivity point to difficulty self-regulating mental resources to align with situational demands—a possible consequence of entrepreneurial stresses. The general parity of scores between established and nascent entrepreneurs suggests ADHD symptoms persist across career maturity levels rather than reflecting transient behaviors. This highlights needs to support focus, impulse control, and stress resilience across all business growth stages. Analyses of differences in mean subgroup symptom rates, correlations with well-being, and predictive modeling based on age, gender, and pandemic impacts could further direct tailored interventions.

These patterns within both attention deficit and hyperactivity domains provide specific insights into how ADHD symptoms manifest for entrepreneurs and identify priority areas for support^31^. The findings confirm ADHD as a persistent set of neurological variances with widespread impacts on management ability. Carefully designed assistance alleviating cognitive load, improving organization, providing structure, and managing restlessness and distraction could empower dyslexic founders to unlock their visionary potential. Moreover, these exploratory regressions provide valuable initial evidence that sleep hygiene and religious activity may be particularly salient for established entrepreneurs’ attention issues. For nascent owners, sleep has an especially strong association with AD symptoms. All entrepreneurs should minimize alcohol intake. While not definitive, these analyses can direct constructive lifestyle supports and prompt further investigation with expanded data. However, the absence of statistical power for many coefficients implies that lifestyle and health behaviors likely only partially contribute to entrepreneurial AD/HD outcomes^32^. Underlying biological factors, genetics, cognitive wiring, and other environmental influences may play substantial roles. Additionally, the high relative standard errors indicate considerable unexplained sample variability, reducing precision^33^.

### Dyslexia symptom and wellbeing regression analysis

The provided regression analyses explore connections between lifestyle factors and dyslexia symptom severity in established and nascent entrepreneurs. By modeling “Mild Symptoms = 0” versus “Major Symptoms = 1” as the binary outcome, the goal is to elucidate potentially modifiable behaviors that may mitigate or worsen reading/writing challenges. This knowledge can inform supportive interventions to bolster resilience and well-being for dyslexic business owners facing pandemic-related anxieties.

For established entrepreneurs (Table 5), no lifestyle variables demonstrate statistically significant relationships with major symptomology at the p < 0.05 level. However, some interesting associations emerge. Longer sleep duration exhibits a negative coefficient, implying more rest could alleviate difficulties, although significance is lacking. The positive coefficient for exercise points to potential gains from physical activity, while religion trends negative, suggesting possible mental health benefits. Healthier diets also correlate with fewer symptoms. The significant constant term indicates an underlying baseline symptom level when all other influences equal zero^34^.

**Table 5:** The output of a linear regression model of dyslexia for established group.

Wellbeing variable	Coefficient	Std. error	t-value	P >|t|	95% conf. interval (lower)	95% conf. interval (upper)
Sleep	− 0.083	0.072	− 1.144	0.259	− 0.228	0.063
Exercise	0.160	0.092	1.741	0.089	− 0.026	0.346
Yoga	0.045	0.116	0.383	0.704	− 0.191	0.280
Healthyfood	− 0.121	0.078	− 1.543	0.131	− 0.280	0.038
Religion	− 0.037	0.112	− 0.335	0.740	− 0.263	0.188
Alcohol	− 0.078	0.093	− 0.832	0.411	− 0.266	0.111
Smoke	− 0.008	0.064	− 0.125	0.901	− 0.138	0.122
_cons	0.925	0.237	3.895	0.000	0.445	1.405

Among nascent owners (Table 6), two variables emerge as statistically significant. Religious engagement demonstrates a negative correlation, connecting greater participation with fewer major symptoms. As this group faces foundational business stresses, faith community support could prove protective. Additionally, healthy eating patterns share a significant negative association. As nutrition strongly impacts neurological function, diet quality may mitigate dysfunctional processing. Sleep, yoga, and alcohol use show positive coefficients but lack significance, while smoking trends negative^35^. Again, the significant constant term implies external factors also affect dyslexia susceptibility.

**Table 6:** The output of a linear regression model of dyslexia for nascent group.

Wellbeing variable	Coefficient	Std. error	t-value	P >|t|	95% conf. interval (lower)	95% conf. interval (upper)
Sleep	0.082	0.048	1.709	0.091	− 0.013	0.176
Exercise	0.006	0.054	0.116	0.908	− 0.102	0.114
Yoga	0.066	0.059	1.113	0.269	− 0.052	0.183
Healthyfood	− 0.109	0.050	− 2.180	0.032	− 0.208	− 0.009
Religion	− 0.121	0.060	− 2.006	0.048	− 0.241	− 0.001
Alcohol	0.072	0.064	1.134	0.260	− 0.054	0.198
Smoke	− 0.040	0.052	− 0.752	0.454	− 0.144	0.065
const	0.646	0.187	3.459	0.001	0.274	1.018

With the continuous reference from the subgroups of the dyslexia symptoms data (Table S2), it is not difficult to find out some specific trends in reporting patterns across established and nascent entrepreneurs. While certain challenges are pervasive, prevalence differs across some domains. Some examples as well as their elaboration are indicated as follows:

- Inattention: 60%+ of both groups affirm difficulties with left/right confusion, map reading, reading speed, memory, spelling, handwriting, phone messages, math, and appointments. This indicates widespread executive function challenges even for basic tasks that undermine focus and retention of information. However, fewer nascent founders report issues with telling time (35% vs 63%), dialing phones (21% vs 60%), forms (59% vs 63%), or mixing up numbers (23% vs 58%). This points to possible benefits from greater reliance on digital interfaces.
- Processing fluency: the most widely reported symptoms relate to fluent literacy processing: spelling (66%), handwriting (62%), reading retrieval (61%), slow reading (58%). These fluency issues can slow and frustrate. Writing samples assessed for reading level, reading rate tracking, free-form journaling, and voice memos could illuminate strengths. Text-to-speech/speech-to-text tools may assist.
- Memory consolidation: remembering reading content (60%), appointments (57%), messages (58%) point to memory and recall challenges. External storage aids like notifications, transcripts, recordings, or assistants could scaffold retention.
- Mathematical processing: 63% affirm difficulty with mental math, while 39% had trouble learning times tables. Math anxiety is common, better conquered through pattern recognition and repetition than rote numeric rules. New notation styles, finger counting, and calculator use could support agility.
- Age-specific trends: while phone use, forms, and numbers cause less trouble for nascent entrepreneurs, learning multiplication was far tougher for established groups (64% vs 21%). Education advances may account for this differential.

The results of the analysis provide a nuanced picture of the relationship between lifestyle factors and dyslexia symptoms, patterns in entrepreneurial dyslexia challenges provide guidance for tailored supports. Mentorship, accommodations, tools assistance and external scaffolds could help dyslexic thinkers excel as innovators by playing to their conceptual strengths.and cetyain exploratory regressions provide initial evidence that lifestyle behaviors, especially diet and religious engagement, may influence dyslexic outcomes for entrepreneurs^36^. Similarly, the lack of significant findings for many of the variables suggests that dyslexia's manifestation among entrepreneurs may be influenced by a complex interplay of factors beyond those measured in the study. The significant constant terms in both groups point to the existence of other influential factors not captured by the included variables. For future research, these results underscore the importance of considering a broader array of variables, including psychological, genetic, and environmental factors, that may impact dyslexia symptoms^37^. Moreover, it emphasizes the need for longitudinal studies to understand the causal relationships better and the potential for interventions that could support entrepreneurs with dyslexia, especially in the context of the additional stressors introduced by the COVID-19 pandemic.

### Wellbeing activities analysis

This section examines correlations between engagement in wellbeing activities and overall wellness among Chinese entrepreneurs during the COVID-19 pandemic. Particular focus is given to entrepreneurs exhibiting ADHD and dyslexia symptoms, as these groups faced heightened stresses. As conveyed in Table S3, established entrepreneurs report noticeably higher smoking and drinking frequencies compared to their nascent counterparts. This potentially signals elevated pandemic-related economic strains provoking unhealthy coping outlets among mature business owners. Meanwhile, broader engagement in positive activities like sleep, diet, and exercise remains largely similar across nascent and established cohorts^38^.

Entrepreneurs with ADHD symptoms had lower frequency of enough sleep compared to those without ADHD. However, the results for exercise and healthy food were similar for both groups, while those with ADHD actually engaged in yoga, meditation, and religious behaviors more often. On the other hand, those with ADHD and Dyslexia had a significantly higher frequency of smoking and drinking compared to those without these symptoms, which is consistent with previous research indicating that individuals with ADHD tend to experience more sleep problems and engage in higher rates of smoking and drinking during the pandemic. However, in terms of healthy self-care behaviors, the dyslexia group exhibited quite different patterns from the ADHD group except for sleep. Those with dyslexia symptoms exercised and had healthy food much less than those without. This is consistent with what previous research has found that people with dyslexia tend to take less care of themselves and live a less healthy lifestyle during the pandemic. Regression analyses reveal sleep duration as the sole wellbeing activity with a consistent, statistically significant positive relationship to wellness across all subgroups. Each additional hour of sleep correlates to a 1.27 point increase in wellness score on average (p = 0.044), controlling for education, age, and gender. This reinforces sleep’s essential biological role in cognitive function, mood stability, and growth hormone release to combat disease^39^.

**Figure 3:**
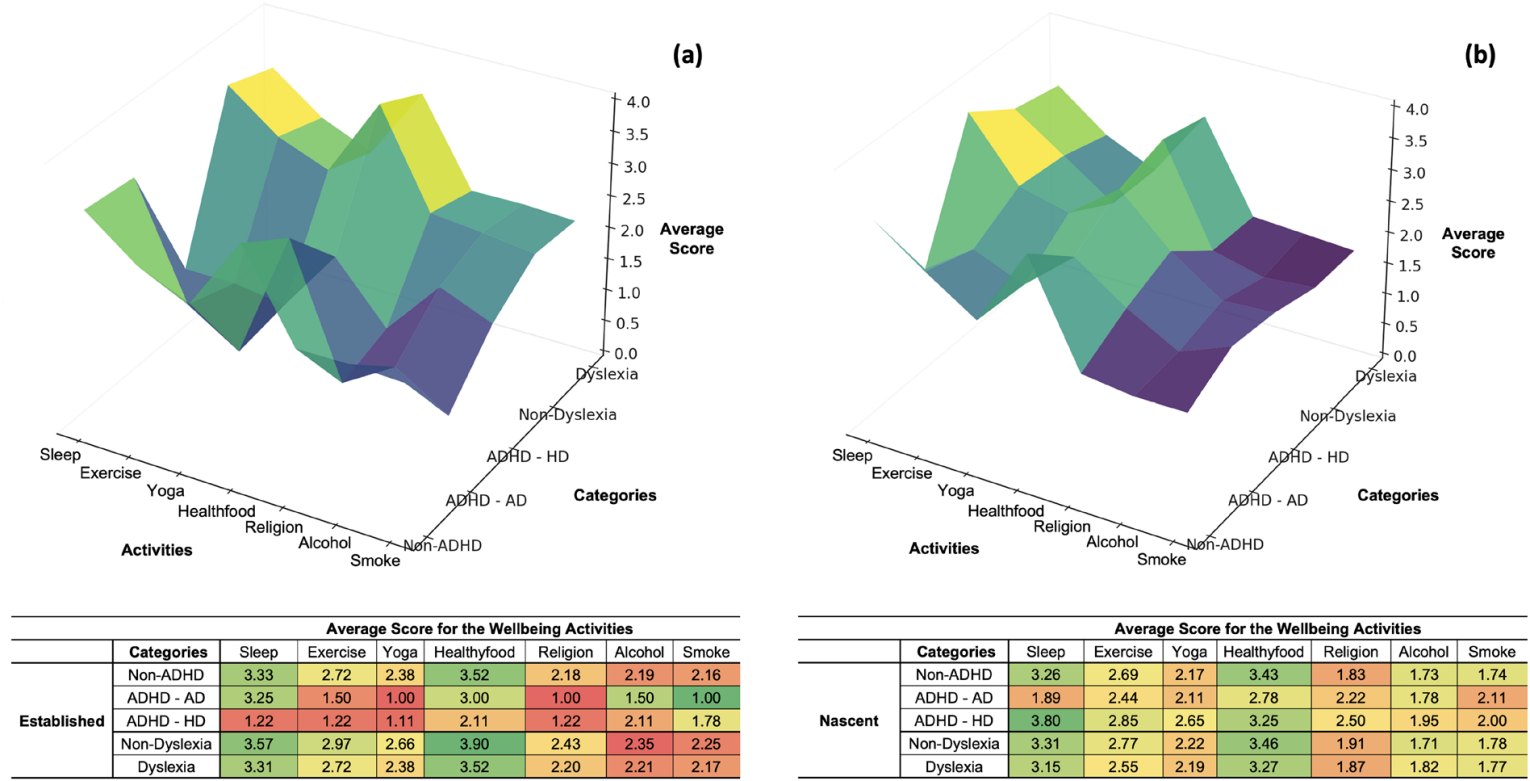
Engagement in wellbeing activities for entrepreneurial groups (**a**) established group and (**b**) nascent group.

Intriguing divergences emerge when comparing entrepreneurs with versus without ADHD or dyslexia, as shown in Fig. 3. The color gradients serve as a visual indication that makes it easy to see which well-being activities each group participates in most and least, which the darker colors indicate higher average scores and lighter colors indicate lower average scores for each well-being activity. For ADHD groups, especially ADHD–HD, while sleep and exercise tend to have reduced participation in these groups, as demonstrated by lighter tones, religion shines out in both panels. This implies that people with ADHD might be more interested in spiritual than in physical activities when it comes to their regimen for overall wellbeing^40^. The observed lower sleep duration and the varied use of substances among certain segments highlight the significance of mental health support in an entrepreneurial setting that meets the unique requirements of its founders. It's interesting to see how established and nascent entrepreneurs prioritize different aspects of well-being. For example, nascent entrepreneurs without ADHD seem to be more interested in health food than established entrepreneurs, which could be a sign of a change in coping mechanisms or way of life as they launch their businesses. In contrast, balanced shades has found for people with dyslexia in both established and nascent groups, reflecting a more even distribution of well-being activities, which could indicate a more consistent daily routine that includes a variety of activities. We also observed that dyslexic entrepreneurs participated in less physical activity and lower consumption of healthy foods compared to non-dyslexic peers. This underscores the necessity of tailored interventions promoting lifestyle balance for those with neurodiverse wiring^41^. While the relationships between wellness and other measured activities remain non-significant in regression modelling, results are likely confounded by overlapping variability from uncaptured pandemic impacts. Further research through longitudinal tracking and more granular stressor data can illuminate more nuanced support strategies for entrepreneurial groups facing external crises. patterns of maintained or heightened engagement on select wellbeing activities among entrepreneurs experiencing pandemic hardship and neurodivergence point to commendable resilience.

Overall, these findings emphasize the possibility for individualized well-being solutions that take into account both the disease (e.g., ADHD, dyslexia) and the entrepreneurial stage (established vs. nascent) in order to successfully promote mental health and overall wellness. It is clear that for both established and nascent entrepreneurs in China, enough sleep has a positive relationship with wellbeing level during the pandemic. While for other variables, the relationship is not very clear, which could be explored further in future studies to better understand the impact of COVID-19 on entrepreneur's wellbeing.

## Differences and similarities between the study's results and those of previous studies in Western Societies

This chapter juxtaposes the findings from our investigation into entrepreneurial wellbeing in China against the backdrop of Western studies, revealing both convergences and divergences in behavior. We integrate a range of scholarly works and recent global wellbeing indexes to enrich the comparative narrative.

### Cross-cultural perspectives on entrepreneurial wellbeing during the pandemic

Our study reveals that established entrepreneurs in China have a higher propensity for smoking and drinking compared to their nascent counterparts—a reversal of the pattern observed in Western societies where the nascent are more prone to these behaviors under the duress of work and stress^1–5,16^. This distinction may reflect cultural variances in coping mechanisms and stress management strategies during the economic perturbations of the pandemic. The comparison between the results of our study and those of previous studies conducted in Western societies reveals some interesting differences and similarities.

#### Differences

1. The study found that established entrepreneurs in China smoked and drank much more than nascent entrepreneurs, which is different from previous studies done in Western societies, where nascent entrepreneurs engaged in unhealthy behaviors more often than established entrepreneurs due to their higher level of stress and commitment in their work.
2. The study found that nascent entrepreneurs in China were more likely to engage in healthy self-care behaviors than established entrepreneurs, which is also different from previous studies done in Western societies^18,19^.
3. The study found that entrepreneurs with ADHD symptoms in China engaged in yoga, meditation, and religious behaviors more often than those without ADHD symptoms, which is different from what previous research has found in Western societies.

#### Similarities

1. The study found that high quality sleep, regular exercise, and healthy food are important for people's mental health and wellbeing, which is consistent with previous research done in Western societies^20^.
2. The study found that entrepreneurs with ADHD symptoms in China were more likely to smoke and drink, which is consistent with what previous research has found in Western societies.
3. The study found that people with dyslexia symptoms in China tended to take less care of themselves and live a less healthy lifestyle, which is consistent with previous research done in Western societies.

It is worth noting that the COVID-19 pandemic has significantly impacted people's mental health and wellbeing worldwide, including in China. The pandemic has resulted in social isolation, economic uncertainty, and increased stress levels, which can lead to mental health problems such as anxiety and depression^14^. Therefore, it is possible that the results of this study may be influenced by the pandemic, as entrepreneurs may have faced additional stress and pressure due to the pandemic's impact on their businesses^18^. In addition, the pandemic may have affected the self-care behaviors of entrepreneurs, as social distancing measures may have limited their ability to engage in healthy behaviors such as exercise or social activities. On the other hand, the pandemic may have also prompted entrepreneurs to prioritize their health and wellbeing, as the importance of self-care has become more evident during the pandemic^30^.

### Divergent behavioral patterns

Our study delineates a clear behavioral divergence between established and nascent entrepreneurs in China. Established entrepreneurs have reported higher frequencies of smoking and alcohol consumption. This pattern deviates from the Western narrative, where it is typically the nascent entrepreneurs who, under the weight of establishing new ventures and the associated stressors, turn more to such unhealthy coping mechanisms^3^. This discrepancy might be influenced by the unique economic pressures exerted by the pandemic on established businesses, possibly exacerbating stress and prompting a turn towards substance use as a relief valve. According to the Global Stress Index (GSI) 2023, these stress-induced behaviors have been especially pronounced due to the economic volatility experienced during the pandemic, suggesting a direct link between business maturity, stress levels, and health behaviors^2,3^.

On the other hand, Chinese nascent entrepreneurs have shown a marked tendency towards healthier self-care practices, which sharply contrasts with the propensity for less healthy behaviors among their Western counterparts. This trend could be signaling a cultural or generational shift towards a more health-conscious approach, perhaps spurred by the global dissemination of wellness knowledge via digital platforms and a growing awareness of the long-term benefits of healthy living^42^. The accessibility of wellness information and resources may have empowered these entrepreneurs to adopt and maintain healthier lifestyles in the face of entrepreneurial challenges. Additionally, the heightened engagement in yoga, meditation, and religious activities among Chinese entrepreneurs with ADHD presents a striking contrast to the lesser utilization of such practices by their Western peers. This could be a reflection of the cultural assimilation of traditional Eastern wellness practices within the entrepreneurial community in China. It may also suggest a diminishing stigma around mental health issues, as these practices are often sought for their mental and emotional benefits. The increased adoption of these holistic practices, which are deeply rooted in Eastern traditions, indicates a cultural synergy that blends age-old techniques with modern entrepreneurial life^1^.

### Convergent wellbeing essentials

Our study reinforces the critical nature of fundamental wellbeing practices that are globally recognized as pillars of mental health: quality sleep, regular physical activity, and balanced nutrition. These essentials of self-care maintain their significance across diverse cultures, echoing the findings of Western studies that underscore their role in sustaining mental health^20^. The pattern of substance use among Chinese entrepreneurs with ADHD mirrors the trends reported on a global scale. This alignment reveals the persistence of challenges in managing ADHD symptoms, regardless of cultural backdrop, and underscores the need for universally accessible support systems tailored to the unique requirements of neurodiverse individuals^43^.

Similarly, Chinese individuals with dyslexia exhibit a tendency towards reduced engagement in self-care activities, a trend that has been documented in various Western societies. This consistency across continents suggests that the lifestyle challenges posed by dyslexia are not constrained by geographic or cultural boundaries. It points to an inherent aspect of the condition that affects lifestyle choices, indicating the necessity for global health strategies that effectively address the specific needs of the dyslexic population^44^. The convergence of these findings highlights the importance of a standardized approach to mental health care that transcends cultural nuances. It calls for a collaborative effort in the global health community to devise strategies that are sensitive to cultural differences while addressing the common needs of individuals with ADHD and dyslexia. Integrating these insights with a comprehensive understanding of cross-cultural practices will be instrumental in promoting optimal mental health and wellbeing for entrepreneurs worldwide.

### The pandemic's universal impact

The COVID-19 pandemic has irrefutably served as a catalyst for widespread mental health challenges. The World Health Organization's in-depth analysis in 2023 underscores the escalation of stress, anxiety, and depression, culminating in what can be described as a global mental health predicament^6^. Entrepreneurs, traditionally viewed as resilient and adaptive, have reported significant alterations in their operational strategies and personal health routines, adapting to the fluctuating business landscape shaped by the pandemic^14,18^. The pervasive impact of the pandemic extends beyond individual health practices to cultural behaviors that dictate self-care among Chinese entrepreneurs. With restrictions on social gatherings and a shift in business communication to virtual platforms, traditional practices such as smoking and drinking in social and business contexts have encountered a transformative shift. The pandemic has potentially recalibrated the approach towards these activities, with a growing emphasis on health and wellness in the entrepreneurial community^44^. This period of global upheaval has illuminated the varying cultural reactions to the stressors of entrepreneurship and wellbeing. While distinct regional responses have emerged, the collective challenges posed by the pandemic are uniformly felt, reinforcing the need for a synchronized international approach to mental health research and policy development.

Our comparative analysis not only illuminates distinct cultural responses to entrepreneurship and wellbeing but also underscores the shared challenges brought forth by the pandemic. This duality of findings prompts a call for continued cross-cultural research, particularly as global societies navigate the post-pandemic landscape.

Subsequent studies should explore the enduring impact of the pandemic on entrepreneurial wellbeing. Longitudinal research will be essential in understanding the evolution of self-care practices and mental health outcomes. Such insights will be critical in shaping culturally sensitive support systems for entrepreneurs worldwide, ensuring that the lessons from this global crisis inform future health and business practices.

## Limitations, recommendations and implication of the findings

### Limitations

Firstly, the time frame for the distribution and collection of survey results was very short due to the time constraint set for this project. Therefore, the total number of responses that could be collected was not very large, and follow-up tests to check reliability could not be conducted. In future research, when enough time is given, more time could be spent on recruiting respondents so that a larger sample size could be achieved to facilitate data analysis. Moreover, follow-up tests and re-tests could also be conducted to ensure the reliability of the survey results.

Secondly, the sources of respondents were limited in terms of background, which could cause a lack of generality. Most of the respondents were from Jiangsu Province, and a significant portion of the participants were current students or alumni from Shanghai Jiaotong University. In future research, the study sample could be generalized to the general population across the country so that the results collected could be more representative of the whole nation.

**Figure 4:**
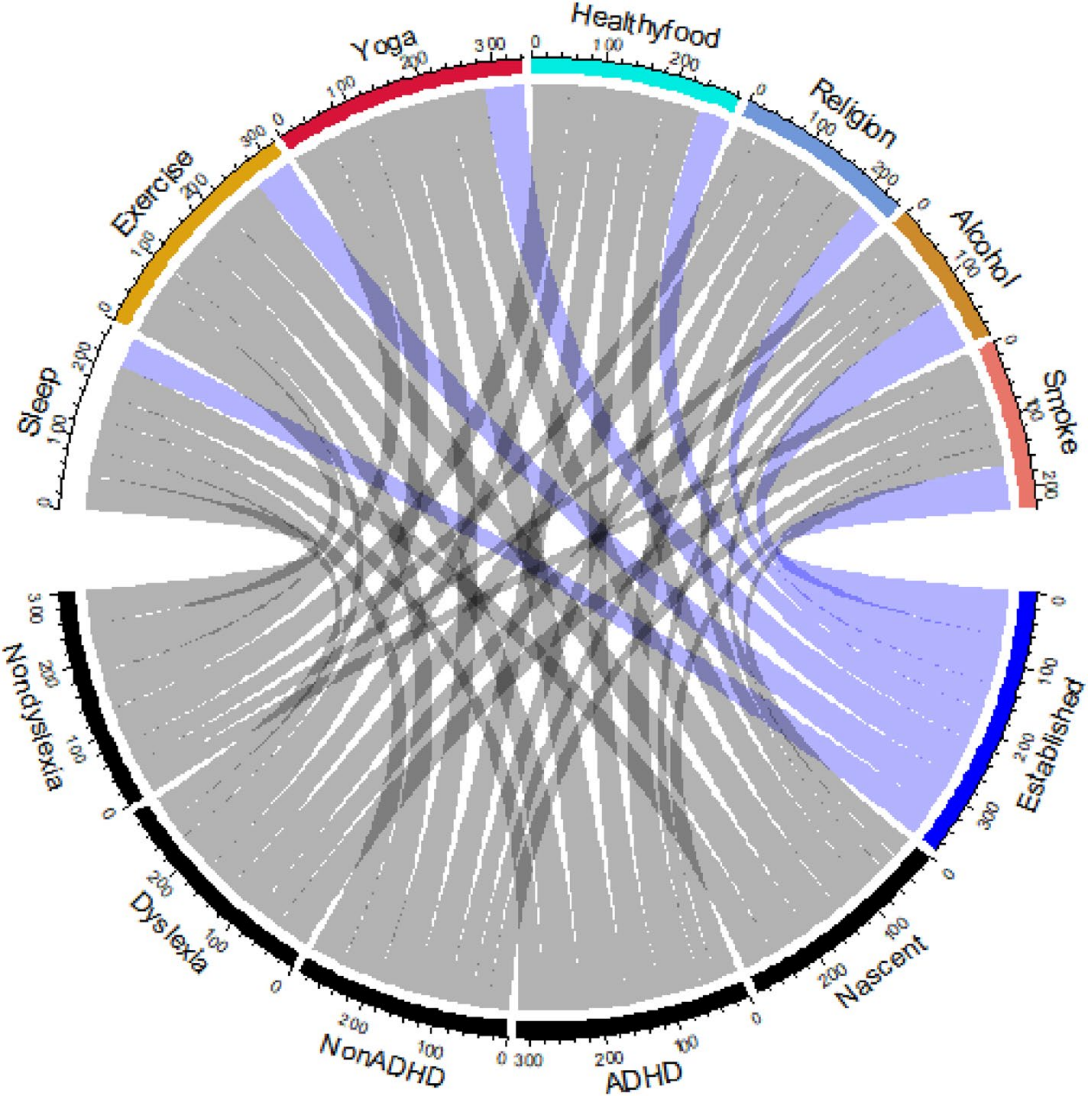
Accordion chart for the comparison between nascent and established entrepreneurs.

Thirdly, the ADHD results obtained from the ASRS-6 measuring scale may not be very reliable, as retests were not conducted for this study to double-check the results due to time constraints. The 18-question version of the same scale could possibly give more accurate results, which could be tried out in future studies. The accordion chart in Fig. 4 appears to be effective in achieving the original vision for this analysis, providing a concise representation of the data compared to scatter plots or linear regression, and even clustering. However, the cluttered appearance of the chart may be attributed to the color scheme and positioning of questions, which could impact visual interpretation. Adjustments such as using more distinct colors for the color bands and optimizing the positioning of questions could improve the overall aesthetics: one advantage of the accordion chart is that it has pre-designed versions and is relatively intuitive to understand^7,19^. However, the most reliable analysis is still the scatter plot, which seems to have been attempted with the original data but not completed. Another alternative approach, similar to a variant of the accordion chart, is a network diagram that represents the relationships between results as nodes connected by lines that indicate the degree of association (e.g., through color or thickness of the lines). This type of network diagram is often used in analyzing search results obtained from several keywords, and it also has a visually pleasing appearance. Nevertheless, it's important to note that the effectiveness of the accordion chart and network diagram may be subjective and dependent on personal preferences. Additionally, other potential limitations may arise from the quality and representativeness of the data used for analysis, as well as the accuracy and rigor of descriptive phrases or terms used for chart labels. Overall, while the accordion chart has its strengths, such as its pre-designed versions and intuitive understanding, it may also have limitations in terms of cluttered appearance and potential subjective interpretations.

Lastly, the COVID-19 pandemic may have also impacted the research and data collection in China, which could have further limitations on the study's results. Due to the pandemic, there may have been limited access to potential respondents, resulting in a smaller sample size than originally planned. Additionally, the pandemic may have affected the mental health of entrepreneurs differently, which could affect the generalizability of the study's findings. It is important to acknowledge the impact of the pandemic on research and data collection and to consider its potential influence on the study's limitations.

### Recommendations

The COVID-19 pandemic has had a significant impact on the entrepreneurial landscape in China, with many start-ups and small businesses struggling to survive due to the economic downturn and restrictions on movement and business operations. As such, it is important for future research to take into account the unique challenges and experiences faced by entrepreneurs during this time^18^.

In addition to the above recommendations, it may also be worthwhile for future research to investigate the impact of the COVID-19 pandemic on the mental health of entrepreneurs in China. The stress and uncertainty associated with the pandemic may exacerbate existing mental health issues and increase the likelihood of new mental health problems arising among entrepreneurs. By understanding the impact of COVID-19 on the mental health of entrepreneurs, appropriate support and interventions can be put in place to promote resilience and well-being in this population^18^.

Last but not the least, there are more areas that could be explored from the surveys. The relationships between ADHD and entrepreneurial performance could be analyzed from the financial information of the businesses and the number of businesses started or owned by the entrepreneurs. This is not discussed in this report as the data collected is very incomplete in this area, due to the reason that many regard financial information as sensitive and private and are unwilling to disclose their statistics in the surveys. Using a much larger study sample could solve this problem so that more effective responses could be collected for the study of this direction. Another area that could be explored is how ADHD symptoms affect entrepreneurs’ relationship and work behaviors with their team members or their employees. Having a deeper understanding of this area would allow people to make better use of those positive aspects and find solutions and remedies for those negative aspects.

### Implications

The findings of this study have significant implications for mental health and entrepreneurship policy in China as well as around the world. The pandemic has led to a surge in mental health problems, including anxiety and depression, among entrepreneurs in China, who are facing unprecedented challenges related to financial instability, supply chain disruptions, and changing consumer behavior. In addition, the pandemic has highlighted the importance of flexibility and adaptability in entrepreneurship, as many businesses have had to pivot their operations to survive during this time. This has created new opportunities for entrepreneurs with ADHD and dyslexia, who may possess unique strengths in innovation and creativity that are well-suited to navigating uncertain and rapidly changing environments.

Therefore, it is crucial for policymakers and mental health professionals in China to take into account the impact of COVID-19 on mental health and entrepreneurship, and to develop policies and programs that address the unique challenges and opportunities faced by entrepreneurs in this context. This could include providing mental health support and resources for entrepreneurs, as well as creating policies that promote greater flexibility and resilience in the entrepreneurial ecosystem.

## Conclusion

The outbreak of COVID-19 in China in late 2019 and early 2020 has had significant impacts on the country's economy and society. The pandemic has led to widespread job losses, business closures, and economic uncertainty, particularly for entrepreneurs and small business owners. The government's response to the pandemic has included measures such as lockdowns, travel restrictions, and social distancing policies, which have further exacerbated the challenges faced by entrepreneurs in China.

The results of this study shed light on the mental health challenges that the Chinese entrepreneurial community faced throughout the pandemic. It shows that entrepreneurs require enhanced mental health support, regardless of their stage of business development. Particularly, it highlights the importance of self-care behaviors, such as sufficient sleep, regular exercise, and healthy diet, in mitigating symptoms of ADHD and Dyslexia, which have been further exacerbated by the pandemic's stresses. The study found that nascent entrepreneurs, who are in the early stages of developing their businesses, have lower levels of wellbeing but engage in healthier self-care behaviors than established entrepreneurs.

Given these findings, the study recommends that mental health support services and resources should be made more widely available to entrepreneurs in China. This could include partnerships with local incubators and entrepreneurship support organizations to provide access to counseling services and stress management workshops. Additionally, policies could be implemented to encourage healthier lifestyle choices and self-care practices among entrepreneurs to improve their wellbeing and overall performance. Overall, the study highlights the importance of addressing mental health concerns in the entrepreneurship community and the need for further research on the topic in China, particularly in the context of the ongoing COVID-19 pandemic.

### Supplementary Information

Supplementary Information.

## Data Availability

The data that support the findings of this study are available from the corresponding author upon reasonable request.
