# Supplementary material for: Entrepreneurial mental health in the wake of COVID-19 in China with an emphasis on attention deficit hyperactivity disorder (ADHD) and dyslexia analysis
Source: Sci Rep. 2024 Mar 19;14:6573. doi: 10.1038/s41598-024-56981-8 (PMC10951316; doi:10.1038/s41598-024-56981-8)
Supplement: Supplementary file 1 — Supplementary Information. [file 41598_2024_56981_MOESM1_ESM.docx]

**Supplementary Information**

**Table S1.** Attention Deficit (AD) and Hyperactivity Disorder (HD) symptom among the entrepreneur subgroups.

| Established entrepreneurs with ADHD (13 out of 173) with 4 AD & 9 HD | Attention Deficit (AD) (4 out of 13) | | | | | | Hyperactivity Disorder (HD) (9 out of 13) | | | | | |
| --- | --- | --- | --- | --- | --- | --- | --- | --- | --- | --- | --- | --- |
|  | Never | Rarely | Sometimes | Often | Very often | Subtotal | Never | Rarely | Sometimes | Often | Very often | Subtotal |
| Trouble wrapping up the final details of a project, once the challenging parts have been done. | 0 | 1 | 2 | 1 | 0 | 4 | 0 | 2 | 3 | 2 | 2 | 9 |
| Difficulty getting things in order when you have to do a task that requires organization. | 1 | 1 | 0 | 1 | 1 | 4 | 1 | 2 | 4 | 2 | 0 | 9 |
| Problems remembering appointments or obligations. | 0 | 0 | 1 | 1 | 2 | 4 | 1 | 1 | 3 | 2 | 2 | 9 |
| Avoid or delay getting started when you have a task that requires a lot of thought. | 1 | 0 | 1 | 1 | 1 | 4 | 1 | 3 | 2 | 2 | 1 | 9 |
| Fidget or squirm with your hands or feet when you have to sit down for a long time. | 1 | 1 | 1 | 0 | 1 | 4 | 0 | 2 | 2 | 3 | 2 | 9 |
| Feel overly active and compelled to do things, like you were driven by a motor. | 0 | 0 | 2 | 2 | 0 | 4 | 1 | 2 | 3 | 3 | 0 | 9 |
|  | | | | | | | | | | | | |
| Nascent entrepreneurs with ADHD (29 out of 244) with 9 AD & 20 HD | Attention Deficit (AD) (9 out of 29) | | | | | | Hyperactivity Disorder (HD) (20 out of 29) | | | | | |
|  | Never | Rarely | Sometimes | Often | Very often | Subtotal | Never | Rarely | Sometimes | Often | Very often | Subtotal |
| Trouble wrapping up the final details of a project, once the challenging parts have been done. | 1 | 2 | 3 | 3 | 0 | 9 | 3 | 5 | 6 | 5 | 1 | 20 |
| Difficulty getting things in order when you have to do a task that requires organization. | 0 | 2 | 2 | 3 | 2 | 9 | 1 | 5 | 5 | 6 | 3 | 20 |
| Problems remembering appointments or obligations. | 0 | 1 | 2 | 4 | 2 | 9 | 2 | 5 | 6 | 4 | 3 | 20 |
| Avoid or delay getting started when you have a task that requires a lot of thought. | 1 | 0 | 3 | 3 | 2 | 9 | 3 | 4 | 4 | 5 | 4 | 20 |
| Fidget or squirm with your hands or feet when you have to sit down for a long time. | 0 | 0 | 4 | 3 | 2 | 9 | 2 | 3 | 5 | 5 | 5 | 20 |
| Feel overly active and compelled to do things, like you were driven by a motor. | 0 | 1 | 3 | 4 | 1 | 9 | 2 | 3 | 4 | 5 | 6 | 20 |

**Table S2.** Dyslexia symptoms among the entrepreneur subgroups.

| Established entrepreneurs with Dyslexia (48 out of 173) | Established Dyslexia | | |
| --- | --- | --- | --- |
|  | Yes | No | Subtotal |
| Do you find difficulty in telling left from right? | 30 | 18 | 48 |
| Is map reading or finding your way to a strange place confusing? | 27 | 21 | 48 |
| Do you take longer than you should to read a page of a book? | 20 | 28 | 48 |
| Do you find it difficult to remember the meaning of what you have read? | 24 | 24 | 48 |
| Do you dislike reading long books? | 32 | 16 | 48 |
| Is your spelling poor? | 33 | 15 | 48 |
| Is your handwriting difficult to read? | 30 | 18 | 48 |
| Do you find it difficult to take messages on the telephone and pass them on correctly? | 31 | 17 | 48 |
| Do you find it difficult to do sums in your head without using your fingers or paper? | 28 | 20 | 48 |
| When using the telephone, do you tend to get the numbers mixed up when you dial? | 29 | 19 | 48 |
| Do you mix up dates and times and miss appointments? | 25 | 23 | 48 |
| Do you find forms difficult and confusing? | 30 | 18 | 48 |
| Do you mix up bus numbers like 95 and 59? | 20 | 28 | 48 |
| Did you find it hard to learn your multiplication tables at school? | 30 | 18 | 48 |
|  | | | |
| Nascent entrepreneurs with with Dyslexia (85 out of 244) | Nascent Dyslexia | | |
|  | Yes | No | Subtotal |
| Do you find difficulty in telling left from right? | 54 | 31 | 85 |
| Is map reading or finding your way to a strange place confusing? | 40 | 45 | 85 |
| Do you take longer than you should to read a page of a book? | 52 | 33 | 85 |
| Do you find it difficult to remember the meaning of what you have read? | 57 | 28 | 85 |
| Do you dislike reading long books? | 60 | 25 | 85 |
| Is your spelling poor? | 56 | 29 | 85 |
| Is your handwriting difficult to read? | 55 | 30 | 85 |
| Do you find it difficult to take messages on the telephone and pass them on correctly? | 63 | 22 | 85 |
| Do you find it difficult to do sums in your head without using your fingers or paper? | 64 | 21 | 85 |
| When using the telephone, do you tend to get the numbers mixed up when you dial? | 30 | 55 | 85 |
| Do you mix up dates and times and miss appointments? | 44 | 41 | 85 |
| Do you find forms difficult and confusing? | 50 | 35 | 85 |
| Do you mix up bus numbers like 95 and 59? | 20 | 65 | 85 |
| Did you find it hard to learn your multiplication tables at school? | 18 | 67 | 85 |

**Table S3.** Average score for the wellbeing activities.

|  |  | **Sleep** | **Exercise** | **Yoga** | **Healthyfood** | **Religion** | **Alcohol** | **Smoke** |
| --- | --- | --- | --- | --- | --- | --- | --- | --- |
| **Established** | Non-ADHD | 3.33 | 2.72 | 2.38 | 3.52 | 2.18 | 2.19 | 2.16 |
|  | ADHD - AD | 3.25 | 1.50 | 1.00 | 3.00 | 1.00 | 1.50 | 1.00 |
|  | ADHD - HD | 1.22 | 1.22 | 1.11 | 2.11 | 1.22 | 2.11 | 1.78 |
|  | Non-Dyslexia | 3.57 | 2.97 | 2.66 | 3.90 | 2.43 | 2.35 | 2.25 |
|  | Dyslexia | 3.31 | 2.72 | 2.38 | 3.52 | 2.20 | 2.21 | 2.17 |
| **Nascent** | Non-ADHD | 3.26 | 2.69 | 2.17 | 3.43 | 1.83 | 1.73 | 1.74 |
|  | ADHD - AD | 1.89 | 2.44 | 2.11 | 2.78 | 2.22 | 1.78 | 2.11 |
|  | ADHD - HD | 3.80 | 2.85 | 2.65 | 3.25 | 2.50 | 1.95 | 2.00 |
|  | Non-Dyslexia | 3.31 | 2.77 | 2.22 | 3.46 | 1.91 | 1.71 | 1.78 |
|  | Dyslexia | 3.15 | 2.55 | 2.19 | 3.27 | 1.87 | 1.82 | 1.77 |
